# Beyond symptom change: a qualitative descriptive study of reported quality of life experiences among adults prescribed medicinal cannabis for anxiety disorders

**DOI:** 10.1007/s11096-026-02176-2

**Published:** 2026-06-09

**Authors:** Leah Roberts, Craig Cumming, Charley A. Budgeon, David B. Preen, Kenneth Lee

**Affiliations:** 1 School of Population and Global Health, University of Western Australia, Perth, Australia; 2 School of Allied Health, University of Western Australia, Perth, Australia

**Keywords:** Anxiety disorders, Medical marijuana, Quality of life, Qualitative research

## Abstract

**Introduction:**

Anxiety disorders are among the most prevalent and disabling mental health conditions and are associated with impairments in quality of life (QoL). Previous studies have examined medicinal cannabis for anxiety and QoL using short-term follow-up or cross-sectional surveys. However, there is little longitudinal qualitative research examining how patients experience QoL changes.

**Aim:**

To explore the perceived impacts on QoL for people with diagnosed anxiety disorders over the first six to twelve months after commencement of medicinal cannabis treatment.

**Method:**

A qualitative descriptive study was conducted using semi-structured interviews and participant diary entries. Adults with an anxiety disorder who had initiated medicinal cannabis within the previous six months were purposively recruited from clinics across Australia. Diary entries were completed at three and six months to capture evolving treatment experiences, with semi-structured interviews conducted within 12 months of treatment commencement. Data were analysed inductively using reflexive thematic analysis following Braun and Clarke’s methodology.

**Results:**

Nine participants (median age 36 years) were included, eight completed diary entries and interviews, and one completed interview only. Participants described improvements across QoL domains following initiation of medicinal cannabis. Themes reflected benefits and challenges, including reclaiming agency over anxiety, QoL changes, social and legal concerns, barriers to treatment initiation, and future treatment intentions. Participants reported perceived improvements in sleep, emotional regulation, relationships, motivation, daily functioning, and overall wellbeing. Several participants also described greater engagement with coping strategies and increased participation in social and daily activities. These benefits occurred alongside challenges, including stigma surrounding disclosure of medicinal cannabis treatment and concerns about cannabis driving legislation and roadside drug testing.

**Conclusion:**

Participants perceived medicinal cannabis as a tool for managing anxiety rather than eliminating symptoms, contributing to improved QoL across domains. Findings highlight the importance of considering QoL alongside symptom management and the role of pharmacists and other healthcare professionals in supporting patients through counselling, treatment monitoring, and stigma reduction. There is a need for evidence-informed policy that balances road safety measures while minimising unintended social and legal harms for patients prescribed medicinal cannabis.

**Supplementary Information:**

The online version contains supplementary material available at https://doi.org/10.1007/s11096-026-02176-2.

## Impact statement

This longitudinal qualitative study highlights that people using medicinal cannabis for anxiety disorders perceived improvements in quality of life beyond symptom reduction, including sleep, relationships, and daily functioning. Findings emphasise the important role of pharmacists and other healthcare professionals in patient counselling, treatment monitoring, stigma reduction, and supporting safe, patient-centred anxiety management.

## Introduction

The burden of mental health disorders is well documented, with anxiety identified as one of the most disabling mental disorders and among the top 25 causes of disease burden worldwide [1–5]. Anxiety disorders are widespread, with lifetime prevalence ranging from 13.6 to 28.8% in high-income countries [6]. QoL encompasses physical, psychological, and social domains of health, influenced by an individual’s experiences, beliefs, expectations, and perceptions [7, 8]. Anxiety disorders are associated with substantial impairments in QoL and psychosocial functioning [9–12]. QoL impairments associated with anxiety disorders include reduced self-esteem, poorer physical health, impaired daily functioning, and socioeconomic disadvantage [7, 10, 11, 13–17], with comorbid mental and physical health conditions often compounding these impacts [2, 15]. Anxiety management commonly involves psychotropic medications, such as benzodiazepines or antidepressants, and cognitive behavioural therapies [18]. Although psychotropic medications are effective in reducing anxiety symptoms [19–21], adverse effects including sexual dysfunction (reported in 30–50% of patients treated with selective serotonin reuptake inhibitors), weight gain, dependence, and withdrawal reactions may negatively affect QoL and contribute to treatment discontinuation [22–25]. Cognitive behavioural therapy is also effective, with 49.5% of patients responding post-treatment and 53.6% at follow-up, typically around six months (range one to 84 months) [26–28]. However, uptake of psychological therapies like cognitive behavioural therapy remains limited by barriers including stigma, low perceived need, doubts about effectiveness, cost, and long waiting periods associated with workforce shortages [29–31]. Consequently, as a result of these limitations some individuals may seek alternative treatments, such as medicinal cannabis [32]. Previous quantitative research has reported improvements in QoL following medicinal cannabis treatment for anxiety [33–37]. Arkell et al. reported sustained improvements across physical and mental health domains of the SF-36 Health Survey, a health-related QoL measure [36]. Mean score changes ranged from 6.60 points (95% CI 4.57–8.63) to 18.31 points (95% CI 15.86–20.77) across up to 15 follow-up assessments conducted at average intervals of 44.6 days [36]. Other studies have similarly reported statistically significant and clinically meaningful QoL improvements following medicinal cannabis treatment, including among people with anxiety [33, 38]. Qualitative studies have also reported perceived anxiety symptom relief alongside experiences of stigma associated with treatment [35, 39]. In Australia, legal and regulatory frameworks may further affect treatment engagement, particularly roadside drug testing laws under which individuals prescribed delta-9-tetrahydrocannabinol (THC) products may still face legal penalties regardless of impairment while driving [40–43]. Penalties may include fines, license disqualification, or imprisonment under the Road Traffic Act 1974 Western Australia (WA) [42, 43].

Previous medicinal cannabis research has predominantly focused on post-traumatic stress disorder, with comparatively limited investigation of generalised anxiety disorder and QoL outcomes [34, 44]. As such, gaps remain in our understanding of how medicinal cannabis use affects quality of life across different anxiety subtypes. To date, no published studies have qualitatively examined longitudinal QoL experiences among people with anxiety disorders following initiation of medicinal cannabis, limiting understanding of how perceived benefits and challenges evolve over time.

## Aim

The aim of this study was to explore the perceived impacts on QoL for people with diagnosed anxiety disorders over the first six to twelve months after commencement of medicinal cannabis treatment.

## Method

### Study design

A longitudinal qualitative descriptive study design was employed. Consistent with qualitative descriptive methodology, the aim was to present participants’ experiences without extensive interpretation or theory development [45, 46]. This study was reported in accordance with the Consolidated Criteria for Reporting Qualitative Research (COREQ) checklist [47].

### Participant selection

Purposive sampling was used to recruit adults who self-reported initiating medicinal cannabis for treatment of an anxiety disorder [48]. Sampling aimed to achieve maximum variation in participant demographics and treatment experiences, including age, gender, concurrent treatments, cannabinoid formulations, and patterns of medicinal cannabis use [49]. This strategy was used to capture a diverse range of experiences and perspectives regarding medicinal cannabis treatment for anxiety disorders. Eligible participants were aged 18 years or older, had initiated treatment within the previous six months, and primarily used medicinal cannabis for anxiety disorders including generalised anxiety disorder, social anxiety disorder, panic disorder, or related anxiety disorders. Exclusion criteria included bipolar disorder, schizophrenia, and current illicit (non-prescribed) cannabis use. Snowball sampling was also used; however, all participants were required to meet inclusion criteria.

Participants were recruited through clinicians, in-clinic advertisements, and clinical email networks across four Australian clinics, some of which provided telehealth services nationally. Eligible participants were contacted up to three times before being considered non-responders, as repeated contact may improve participation rates [50]. Despite recruitment efforts, participants were recruited only from WA and New South Wales.

LR, a female PhD student with qualitative interviewing experience and no prior relationship with participants, provided study information where possible. Recruitment was guided by the concept of information power, whereby relevance and richness of data are prioritised over sample size alone, consistent with Braun and Clarke’s reflexive thematic analysis [51, 52]. Given the focused study aim, specific sample, and longitudinal design with multiple data collection points, recruitment continued until the dataset provided sufficient depth, diversity, and richness of accounts to support nuanced thematic interpretation relevant to the research aim [51, 52]. This resulted in a final sample of nine participants.

### Data collection

Eligibility was determined using a pre-screening questionnaire administered via Qualtrics [53], which collected demographic, diagnostic, treatment, and prior medicinal cannabis use information (Supplementary Materials 1).

Data were collected through participant diary entries and semi-structured interviews. Participants were invited to complete diary entries at three and six months following study commencement, either via Qualtrics or by telephone according to participant preference. Entries included prompts regarding completion date, medicinal cannabis products used, and perceived treatment experiences (Supplementary Materials 2).

Semi-structured interviews were conducted by LR within 12 months of treatment commencement, either in person, by telephone, or via Zoom or Microsoft Teams according to participant preference. The interview guide was developed through literature review and internal expert discussion within the research team. It explored experiences prior to treatment, medicinal cannabis use, perceived changes in daily life and functioning, comparisons with other treatments, and future anxiety management plans (Supplementary Materials 3). A pilot interview was conducted to refine the interview guide and interview process.

Written informed consent was obtained prior to participation, with additional verbal consent recorded at interview commencement. Interviews lasted approximately 60 min to allow sufficient time for participants to share their experiences. Interviews were audio-recorded, transcribed using MS Teams or OtterAI software with institutional ethics approval, reviewed against original recordings for accuracy, and de-identified prior to analysis. Transcripts were produced using a clean verbatim approach, and participants were offered the opportunity to review transcripts and select a pseudonym. No repeat interviews were conducted.

### Data analysis

Reflexive thematic analysis was conducted by LR following Braun and Clarke’s methodology [54] using NVivo 12 software [55]. Diary entries and interview transcripts were coded inductively, with codes developed through repeated reading of the data and iteratively refined into themes and subthemes. Consistent with COREQ recommendations, LR reflected on assumptions regarding medicinal cannabis and anxiety shaped by previous research involvement and personal experiences with mental health. LR acknowledged the potential for these perspectives to influence early coding decisions. To support reflexivity and ensure interpretations remained grounded in the data, transcripts were revisited multiple times throughout analysis. LR is an experienced qualitative researcher with a background in journalism, informing a reflexive approach to interviewing and analysis, including active bracketing of personal views and attentiveness to participants’ accounts. Factors such as gender, age, and background were also considered within reflexive practice. An initial codebook developed by LR was reviewed by members of the research team (KL, CC), who examined coding outputs independently. Coding discrepancies and interpretations were discussed within the research team and resolved through consensus.

### Quality assurance

Several strategies were employed to enhance trustworthiness [56, 57]. Confirmability was supported through LR’s reflexive journaling and maintenance of an audit trail documenting coding decisions and theme development. Credibility was enhanced through peer debriefing and ongoing discussion of interpretations within the research team. Transferability was supported through purposive sampling and detailed description of the study context and participant characteristics. Dependability was strengthened through consistent application and review of the coding framework across the analytic process.

### Ethics approval

This study was approved by the University of Western Australia Human Research Ethics Committee (2023/ET000618).

## Results

Nine participants (median age 36 years; six females, three males) were included. Table 1 presents participant characteristics and cannabinoid preparations used. Table 2 provides details on the themes and constituent subthemes.

**Table 1:** Demographic characteristics and medicinal cannabis use information of participants

Participant	Gender	Age	Occupation	Concurrent Psychological Treatment	Primary Cannabinoid Product	Frequency of use	Formulation/route	Dosage
Bloom	Female	45	Family counsellor	No	THC-dominant	3–4 times per week	Oral oil	0.2 mL per dose; THC 20 mg/mL
Candy	Female	39	School officer	No	CBD-dominant (with THC for sleep)	Daily	Oral oil	CBD oil 100 mg/mL (0.5 mL per dose); THC oil 26.3 mg/mL at night
Fennic	Male	32	Student	Yes	THC-dominant	Daily	Oral oil	0.3–0.5 mL per dose; THC 26.3 mg/mL
Sophie	Female	36	Stay-at-home mother/business owner	No	THC-dominant (CBD when needed)	Daily	Oral edible (gummy)	Half a gummy per dose (THC-based; dose not specified)
Jane	Female	31	Marketing	Yes	Mixed THC/ CBD (with THC-dominant use when needed)	2–3 times per week	Oral oil; inhaled dried flower (optional)	Oil: THC 20 mg/mL, CBD 1 mg/mL; flower strength not specified
MGJC	Male	32	Unemployed	No	CBD-dominant + THC-dominant	Daily	Oral capsule; inhaled dried flower	CBD capsule (dose not specified); THC flower ~ 30%
Bob	Male	74	Retired	Yes	CBD-dominant + THC-dominant	Daily	Oral oil; inhaled dried flower	CBD oil (dose not specified); THC flower used at night
Amber	Female	31	Temporary work	Yes	THC-dominant	Daily	Inhaled dried flower	~ 25% THC; ~ 2 g per day
Jemima	Female	62	Retired	Yes	Mixed THC CBD	Daily	Oral oil; oral edible (gummy)	CBD oil 40 mg/mL (full spectrum); gummies 10 mg THC / 10 mg CBD

Participants are identified using self-selected pseudonyms to maintain confidentiality. THC refers to tetrahydrocannabinol and CBD refers to cannabidiol. THC-dominant refers to products with a higher ratio of THC relative to CBD. mg refers to milligrams and mL refers to millilitres. Participant-reported brand names were excluded from the table. Products were classified according to their active cannabinoid content, formulation, and dose (where reported) to improve clarity and comparability. Where precise dosing information was unavailable, this is indicated as “dose not specified”

**Table 2:** Overview of themes and subthemes

Theme	Subthemes
Theme One: Reclaiming Agency Over Anxiety Symptoms	Reduced emotional reactivity and perceived control of anxiety symptoms Improved motivation, mental clarity and engagement with coping strategies
Theme Two: Improved Quality of Life Across Sleep, Relationships, Physical and Spiritual Wellbeing	Restorative Sleep Improved Relationships Greater Physical Wellbeing and Connection to Spirituality
Theme Three: Social, Legal and Safety Concerns Inhibit Disclosure and Confine Patients to their Homes	Stigma of Disclosure Fear of Legal Implications
Theme Four: Lack of Knowledge and Concerns of Side Effects Barrier to Accessing Medicinal Cannabis Treatment	Limited awareness of medicinal cannabis treatment options Stigma as a barrier to treatment initiation
Theme Five: Future Use Reflects Balancing Desire for Symptom Relief and Desire to Reduce Long-Term Use	Satisfaction of current treatment Desire to reduce or discontinue medication as part of broader anxiety management

### Theme One: Reclaiming agency over anxiety symptoms

All participants described a shift in their relationship with anxiety following initiation of medicinal cannabis, characterised by greater emotional regulation, reduced reactivity, and increased perceived control. Prior to treatment, anxiety was described as overwhelming and difficult to manage, affecting daily functioning, relationships, and employment. Bob (aged 74, interview) recalled:

“I was a real mess [pause] I didn’t know what to do [pause] I couldn’t do any of it, it was hard to go out or do anything.”

Participants also described heightened emotional reactivity prior to treatment. Candy (aged 39, interview) stated:

“It was just always stressful […] kids would do something, and I would just fly off the handle.”

Following treatment initiation, participants commonly described anxiety as remaining present but becoming more manageable. In her three-month diary entry, Jemima (aged 62) described feeling “more relaxed” and “less reactive.” While Jane (aged 31, six-month diary entry) wrote that although her mental health “is not perfect,” she was “functioning much better than ever anticipated.” Fennic (aged 32, interview) explained:

“It’s not like a miracle. It's not like it gets rid of it completely. It's still there and I still very much feel it. It's just that I think I have more of an emotional and mental control over it.”

Some participants described being better able to engage with psychological coping strategies alongside medicinal cannabis treatment. Fennic (aged 32, three-month diary entry) described using breathing exercises to prevent acute anxiety attacks, while Bob (aged 74, interview) described using cognitive reframing during periods of panic:

“That it’ll pass you know… the panic attacks or anxiety it’ll pass, and I’ll be alright sort of thing.”

Participants also described greater motivation, mental clarity, and engagement in daily activities following treatment initiation. Several participants linked these changes to reduced anxiety-related mental burden and greater “headspace.” Jemima (aged 62, interview) stated:

“There’s so much I need to get done and the difference is now I actually have the motivation and the headspace to do it.”

### Theme Two: improved quality of life across sleep, relationships, physical and spiritual wellbeing

Beyond anxiety symptom changes, participants described perceived improvements across sleep, relationships, physical wellbeing, and spirituality.

#### Restorative sleep

Prior to treatment, participants described disrupted sleep characterised by difficulty falling asleep, waking during the night, and waking with heightened anxiety. Following treatment initiation, eight participants described improved sleep quality, including deeper and more restorative sleep. Bob (aged 74, interview) described sleep disruption due to panic attacks prior to treatment. Following treatment, he stated:

“Sleep is better and when I wake up, I get up better in the morning.”

Participants commonly linked improved sleep with greater energy, emotional regulation, and improved anxiety management. Jemima (aged 62, interview) explained:

“When you have a good night’s sleep, you are a different person really.”

One exception was identified, with MGJC (aged 32, interview) reporting no improvement in sleep due to concurrent medications.

### Improved relationships

Participants described improved anxiety management as positively affecting interpersonal relationships and social engagement. Jemima (aged 62, three-month diary entry) stated:

“I am able to engage with friends and family more calmly.”

Amber (aged 31, interview) similarly described improved emotional regulation during social interactions:

“It actually helps me not only tolerate other people around me, but me be a more tolerable person.”

Participants who were parents commonly described greater patience with their children. Sophie (aged 36, interview) explained:

“I still can get cranky with them, but the reaction is tempered to the realms of what they deserve.”

### Physical and spiritual wellbeing

Participants also described perceived improvements in physical wellbeing, which they associated with greater “mental space” and reduced psychological burden. Candy (aged 39, interview) described returning to the gym after treatment initiation, while Jemima (aged 62, interview) stated:

“I just feel really well-oiled like I’ve been to the mechanics or something.”

Some participants also described greater feelings of meaning, connection, and spirituality. Sophie (aged 36, interview) described feeling “more spiritually guided… just happier,” and characterised treatment as giving her “a new lease on life.”

### Theme Three: social, legal and safety concerns inhibit disclosure and confine patients to their homes

Despite perceived benefits, participants described ongoing stigma and legal concerns associated with medicinal cannabis treatment. Many described reluctance to disclose treatment due to fears of judgement and negative stereotypes associated with recreational cannabis use. Bob (aged 74, interview) stated:

“Because it’s an ‘illicit drug’… they think you're just a druggie sort of thing, you know. But that's not the way it is with me.”

Participants commonly distinguished prescribed medicinal cannabis from recreational cannabis use, emphasising therapeutic intent and responsible use. Jane (aged 31, interview) reflected on peers’ surprise regarding her treatment:

“I’ve interpreted that as oh, she's not like a stoner. She's still doing life.”

Stigma was also described in interactions with some health professionals and influenced disclosure decisions involving family, peers, and clinicians. Participants additionally described anxiety relating to roadside drug testing and driving legislation in WA and NSW. Concerns included losing independence, employment, or the ability to respond to emergencies despite efforts to avoid driving while impaired. Bob (aged 74, interview) described feeling confined by driving restrictions:

“I just feel a bit confined… that gets me really anxious and panicky.”

Participants commonly expressed frustration that legislation did not distinguish between impairment and the presence of THC in the body. Jane (aged 31, interview) stated that being able to declare her prescription during roadside testing would substantially reduce her anxiety.

### Theme Four: limited knowledge and concerns about side effects delayed treatment access

Participants described limited knowledge of medicinal cannabis as a barrier to treatment access. Candy (aged 39, interview) stated she had been unaware of cannabidiol (CBD) oil and would have sought treatment earlier had she known more about it. Concerns regarding side effects also contributed to initial hesitancy, particularly fears of cognitive impairment. Fennic (aged 32, interview) described uncertainty regarding potential long-term cognitive effects.

Following treatment initiation, participants commonly reported minimal side effects and several expressed wishing they had commenced treatment earlier. Bloom (aged 45, interview) described feeling “more sleepy,” while Candy (aged 39, interview) described occasionally feeling “a little high” when taking excessive THC, although she described this as “very minimal.” Bob (aged 74, interview) stated:

“I can function normally, the memory’s good… it doesn’t impact me in that way.”

Stigma was also described as a barrier to treatment access. Bob (aged 74, interview) expressed concerns regarding being labelled a “druggie,” while Bloom (aged 45, interview) contrasted medicinal cannabis with alcohol, describing cannabis use as more socially “frowned upon.” Participants additionally described positive experiences among family members or friends as encouraging consideration of medicinal cannabis treatment.

### Theme Five: future use reflected balancing symptom relief and reducing long-term reliance

All participants expressed satisfaction with medicinal cannabis treatment and willingness to continue use in the short term. However, longer-term intentions varied, with several participants describing a desire to reduce or discontinue treatment to avoid medication reliance and strengthen self-management strategies. Amber (aged 31, interview) described reducing her dose alongside psychological support:

“To be more mentally strong, so that I don’t find that I need it anymore.”

Only one participant expressed concerns regarding potential long-term cognitive effects related to a family history of Alzheimer’s disease. Other participants were comfortable continuing medicinal cannabis alongside psychological therapies, commonly framing treatment as one component of broader anxiety management. Jane (aged 31, interview) explained:

“I think it’s always going to be part of the kind of mix… whether it’s the primary kind of treating medication or not, that doesn’t really matter.”

Overall, participants described willingness to continue medicinal cannabis long term if required for ongoing anxiety management.

## Discussion

This study explored QoL experiences among people living with anxiety disorders during the year following initiation of medicinal cannabis treatment. Participants described perceived improvements across QoL domains including emotional regulation, sleep, relationships and daily functioning. Medicinal cannabis was framed not as a “cure” for anxiety, but as a management tool supporting greater agency, emotional regulation, and engagement in everyday life. These perceived benefits coexisted with ongoing stigma and roadside drug testing.

Participants’ accounts align with previous quantitative and qualitative studies reporting improvements in QoL and anxiety symptoms [33, 35, 36, 58] alongside ongoing experiences of stigma following treatment initiation [35, 39]. To date, no published studies have qualitatively examined longitudinal QoL experiences among people with anxiety disorders following initiation of medicinal cannabis; this study addresses that gap.

### Medicinal cannabis as a management tool rather than a “cure”

Findings from this study suggest medicinal cannabis was perceived less as a cure for anxiety and more as a tool for symptom management. Across the study, anxiety was described as remaining present but less intense and overwhelming, particularly during acute episodes where participants reported greater self-regulation and perceived control. This is consistent with previous research reporting reductions in anxiety symptoms following medicinal cannabis treatment [59–62]. However, unlike previous literature primarily focused on symptom reduction, this study identified broader QoL and recovery-orientated benefits, including greater self-efficacy, agency, and capacity to engage in daily life alongside ongoing symptoms. Medicinal cannabis was perceived to facilitate engagement with coping strategies, including breathing exercises and cognitive reframing, that had previously felt inaccessible. This aligns with contemporary recovery-orientated approaches in mental health, which emphasise meaningful functioning and self-management alongside ongoing symptoms [12, 63, 64], with medicinal cannabis positioned as one component of a broader therapeutic “toolbox.”

Participants who engaged in psychological interventions alongside medicinal cannabis treatment described complementary but distinct roles, with medicinal cannabis perceived to reduce emotional overwhelm and facilitate engagement with therapeutic coping strategies. While studies suggest the combination of treatments is beneficial for post-traumatic stress disorder [64, 65], evidence remains limited for general anxiety disorder. Our findings provide preliminary insights into how medicinal cannabis may be used alongside psychological therapies as part of broader anxiety management.

This study found participants described a desire to eventually reduce or discontinue medicinal cannabis use, reflecting preferences for autonomy and reduced medication reliance rather than dissatisfaction with treatment itself. Similar concerns regarding long-term medication use and dependence have been reported in previous literature [65–67]. However, participants remained willing to continue treatment if needed, suggesting perceived benefits outweighed preferences to discontinue. Reported adverse effects were typically mild and not perceived to interfere substantially with daily functioning, despite previous literature identifying potential risks including worsening anxiety symptoms and cognitive decline [68–70].

### Re-engagement with life

Findings from this study suggest improvements in anxiety symptoms may support broader engagement with everyday life across social, occupational, relational, and physical domains. Sleep was among the most consistently valued changes, with improved sleep quality commonly associated with greater energy, emotional regulation, and daily functioning. These findings are consistent with broader literature [35, 36, 58]. Furthermore, poor sleep quality and disturbances are linked to impairments in cognitive, emotional, and interpersonal functioning, contributing to the onset and maintenance of anxiety disorders [71, 72], and highlighting the importance of addressing sleep in anxiety management [73, 74].

Anxiety disorders are commonly associated with social withdrawal and interpersonal difficulties [10, 75, 76]. Findings from this study described improved interpersonal relationships, greater emotional availability, and increased willingness to participate in social activities. While previous questionnaire-based studies have identified improvements in relationship-related QoL domains [36, 58], this study provides insight into how participants interpreted these changes within everyday life contexts.

Findings from this study suggest improvements in anxiety management extended beyond symptom relief to occupational and physical domains, including greater engagement with work, concentration, motivation, exercise, and daily routines. Our results are consistent with questionnaire-based studies reporting improvements in physical health-related quality of life following medicinal cannabis treatment [36, 58]. Participants in this study attributed increased physical activity and functioning to reduced psychological burden and greater capacity to engage in previously unattainable routines.

### Stigma and legal concerns as a source of ongoing anxiety

Despite perceived therapeutic benefits, stigma and driving-related legal concerns remained persistent sources of anxiety. In this study, participants frequently distinguished prescribed medicinal cannabis from recreational cannabis use, framing treatment in terms of legitimacy, therapeutic intent, and responsible use while also describing concerns regarding judgement and social perceptions of treatment. Similar findings have been reported among people prescribed medicinal cannabis for chronic pain and other conditions [35, 39, 77, 78].

Roadside drug testing emerged as a significant source of ongoing anxiety within this study. Concerns regarding licence loss, employment and caregiving consequences, and restrictions on independence despite efforts to avoid driving while impaired were found. These findings have also been reported for patients prescribed medicinal cannabis for endometriosis [79]. Emerging evidence suggests THC-related driving impairment is complex and influenced by factors including timing, dose, route of administration, and individual variability, with impairment not reliably inferred from product strength alone [80–82]. However, current Australian roadside drug testing approaches, including under the Road Traffic Act 1974, primarily detect the presence of THC rather than impairment itself [42, 43]. Consequently, patients prescribed THC-based medicinal cannabis may still face criminal penalties despite not driving while impaired, contributing to psychological distress and practical consequences reflected in participants’ accounts. Findings from this study support growing calls for impairment-based legislative approaches; however, no ‘gold standard’ measure of cannabis-related driving impairment currently exists. Ongoing research by the Victorian state government aims to better understand impairment thresholds and inform more nuanced policy responses [83]. These findings highlight the need for evidence-informed policy that maintains road safety while minimising unintended social and legal harms for patients prescribed medicinal cannabis.

### Clinical and practice implications

Findings from this study have implications for pharmacists and other healthcare professionals involved in anxiety management. As accessible healthcare professionals, pharmacists are often involved in counselling regarding medicinal cannabis administration, adverse effects, drug interactions, safe use, and treatment monitoring [84, 85]. Consistent with broader literature emphasising QoL alongside symptom management [12, 33, 63, 64], findings also highlight ongoing stigma surrounding medicinal cannabis treatment, suggesting pharmacists may help reduce stigma through evidence-informed counselling and patient education [84].

### Future research

Future research could examine QoL outcomes across broader demographic, cultural, and clinical contexts and explore how experiences evolve over longer periods of medicinal cannabis treatment. Further work is also needed to develop and validate approaches to assessing driving impairment following prescribed medicinal cannabis treatment to support road safety and inform policy.

### Strengths and limitations

A key strength of this study was the longitudinal design, which addressed a gap in the literature by qualitatively examining quality of life experiences among people with anxiety over a period of 12 months after treatment commencement. While existing research has largely relied on quantitative registry data [33, 35, 36, 58], this study provides in-depth, lived-experience accounts of perceived QoL changes. The use of both diary entries and semi-structured interviews enabled contextualised insight into participants’ day-to-day experiences and allowed for reflection on change over time.

This study has several limitations. As a qualitative descriptive study using reflexive thematic analysis, the findings were not intended to objectively verify participants’ accounts or establish causal relationships, but rather to provide in-depth insights into how participants made sense of their experience with medicinal cannabis treatment over time.

While several strategies were employed to enhance trustworthiness, limitations remain. Credibility may have been influenced by expectancy effects, placebo-related influences, recall bias, evolving understandings of treatment, and social desirability bias, particularly given ongoing stigma surrounding cannabis use. No objective quality of life measures were collected within this qualitative component, and participants willing to participate may have held more favourable views towards medicinal cannabis treatment. To mitigate these limitations, we engaged with several clinics across Australia and offered participants multiple ways of being interviewed (both online and in person) to maximise opportunities for participation. To reduce social desirability bias, participants were offered anonymised diary entries in addition to interviews.

Although confirmability was strengthened through reflexive journalling, peer debriefing, and maintenance of an audit trail, it was not possible to disentangle treatment-related changes from contextual factors such as life events or concurrent psychological therapies. Transferability may also be limited as the study involved a small purposively sampled cohort within the Australian regulatory context, where state-based legal frameworks may uniquely shape experiences of anxiety, stigma, and QoL. Although maximum variation purposive sampling was used, the sample lacked cultural and linguistic diversity, with no culturally and linguistically diverse participants included. This limits insight into how cultural beliefs, stigma, and social attitudes towards cannabis may shape experiences [86].

## Conclusion

This longitudinal qualitative descriptive study explored perceived QoL impacts among people with anxiety disorders during the first six to 12 months of medicinal cannabis treatment. Participants described perceived improvements in emotional regulation, sleep, relationships, daily functioning, and engagement in meaningful activities, while framing medicinal cannabis as a management tool rather than a cure for anxiety. However, these perceived benefits coexisted with ongoing stigma and driving-related legal concerns. Findings highlight the importance of considering QoL alongside symptom management and the role of pharmacists and other healthcare professionals in supporting patients through counselling, treatment monitoring, and stigma reduction. Further longitudinal research is needed to better understand long-term QoL outcomes and inform clinical practice and policy.

## Supplementary Information

Below is the link to the electronic supplementary material.

Supplementary file1 (DOCX 24 kb)

Supplementary file2 (DOCX 389 kb)

Supplementary file3 (DOCX 17 kb)

## Data Availability

No datasets were generated or analysed during the current study.

## References

[1] Vos T, Lim SS, Abbafati C, et al. Global burden of 369 diseases and injuries in 204 countries and territories, 1990–2019: a systematic analysis for the Global Burden of Disease Study 2019. The Lancet. 2020;396(10258):1204–22. 10.1016/S0140-6736(20)30925-9. PMC7567026 33069326

[2] Surtees PG, Wainwright NWJ, Khaw K-T, et al. Functional health status, chronic medical conditions and disorders of mood. Br J Psychiatry. 2003;183(4):299–303. 10.1192/bjp.183.4.299. 14519607

[3] Kessler RC, Petukhova M, Sampson NA, et al. Twelve‐month and lifetime prevalence and lifetime morbid risk of anxiety and mood disorders in the United States. Int J Methods Psychiatr Res. 2012;21(3):169–84. 10.1002/mpr.1359. 22865617 PMC4005415

[4] Ferrari AJ, Santomauro DF, Aali A, et al. Global incidence, prevalence, years lived with disability (YLDs), disability-adjusted life-years (DALYs), and healthy life expectancy (HALE) for 371 diseases and injuries in 204 countries and territories and 811 subnational locations, 1990–2021: a systematic analysis for the Global Burden of Disease Study 2021. The Lancet. 2024;403(10440):2133–61. 10.1016/S0140-6736(24)00757-8. PMC11122111 38642570

[5] Hay SI, Ong KL, Santomauro DF, et al. Burden of 375 diseases and injuries, risk-attributable burden of 88 risk factors, and healthy life expectancy in 204 countries and territories, including 660 subnational locations, 1990–2013;2023: a systematic analysis for the Global Burden of Disease Study 2023. The Lancet. 2025;406(10513):1873–922. 10.1016/s0140-6736(25)01637. PMC12535840 41092926

[6] Michael T, Zetsche U, Margraf J. Epidemiology of anxiety disorders. Psychiatry. 2007;6(4):136–42.

[7] Testa MA, Simonson DC. Assessment of quality-of-life outcomes. N Engl J Med. 1996;334(13):835–40. 10.1056/NEJM199603283341306. 8596551

[8] Jones P, Drummond PD. A summary of current findings on quality of life domains and a proposal for their inclusion in clinical interventions. Front Psychol. 2021;12:747435. 10.3389/fpsyg.2021.747435. 34777139 PMC8586497

[9] Mendlowicz MV, Stein MB. Quality of life in individuals with anxiety disorders. Am J Psychiatry. 2000;157(5):669–82. 10.1176/appi.ajp.157.5.669. 10784456

[10] Olatunji BO, Cisler JM, Tolin DF. Quality of life in the anxiety disorders: a meta-analytic review. Clin Psychol Rev. 2007;27(5):572–81. 10.1016/j.cpr.2007.01.015. 17343963

[11] Wilmer MT, Anderson K, Reynolds M. Correlates of quality of life in anxiety disorders: review of recent research. Curr Psychiatry Rep. 2021;23(11):77. 10.1007/s11920-021-01290-4. 34613508 PMC8493947

[12] Hohls JK, König H-H, Quirke E, et al. Anxiety, depression and quality of life—a systematic review of evidence from longitudinal observational studies. Int J Environ Res Public Health. 2021;18(22):12022. 10.3390/ijerph182212022. 34831779 PMC8621394

[13] Henning ER, Turk CL, Mennin DS, et al. Impairment and quality of life in individuals with generalized anxiety disorder. Depress Anxiety. 2007;24(5):342–9. 10.1002/da.20249. 17091478

[14] Barrera TL, Norton PJ. Quality of life impairment in generalized anxiety disorder, social phobia, and panic disorder. J Anxiety Disord. 2009;23(8):1086–90. 10.1016/j.janxdis.2009.07.011. 19640675 PMC2782397

[15] Ruscio AM, Hallion LS, Lim CCW, et al. Cross-sectional comparison of the epidemiology of DSM-5 generalized anxiety disorder across the globe. JAMA Psychiat. 2017;74(5):465–75. 10.1001/jamapsychiatry.2017.0056. PMC5594751 28297020

[16] Hoffman DL, Dukes EM, Wittchen HU. Human and economic burden of generalized anxiety disorder. Depress Anxiety. 2008;25(1):72–90. 10.1002/da.20257. 17146763

[17] Baxter AJ, Vos T, Scott KM, et al. The global burden of anxiety disorders in 2010. Psychol Med. 2014;44(11):2363–74. 10.1017/S0033291713003243. 24451993

[18] Bystritsky A. Treatment-resistant anxiety disorders. Mol Psychiatry. 2006;11(9):805–14. 10.1038/sj.mp.4001852. 16847460

[19] Ravindran LN, Stein MB. The pharmacologic treatment of anxiety disorders: a review of progress. J Clin Psychiatry. 2010;71(7):839–54. 10.4088/JCP.10r06218blu. 20667290

[20] Jakubovski E, Johnson JA, Nasir M, et al. Systematic review and meta-analysis: dose–response curve of SSRIs and SNRIs in anxiety disorders. Depress Anxiety. 2019;36(3):198–212. 10.1002/da.22854. 30479005

[21] van der Linden GJH, Stein DJ, van Balkom AJLM. The efficacy of the selective serotonin reuptake inhibitors for social anxiety disorder (social phobia): a meta-analysis of randomized controlled trials. Int Clin Psychopharmacol. 2000;15:S15–23. 10.1097/00004850-200008002-00004. 11110015

[22] Zhao F-Y, Kennedy GA, Xu P, et al. Identifying complementary and alternative medicine recommendations for anxiety treatment and care: a systematic review and critical assessment of comprehensive clinical practice guidelines. Front Psychiatry. 2023;14:1290580. 10.3389/fpsyt.2023.1290580. 38152358 PMC10751921

[23] Sarris J, Moylan S, Camfield DA, et al. Complementary medicine, exercise, meditation, diet, and lifestyle modification for anxiety disorders: a review of current evidence. Evid Based Complement Alternat Med. 2012;2012(1):809653. 10.1155/2012/809653. 22969831 PMC3434451

[24] Hirschfeld RMA. Long-term side effects of SSRIs: sexual dysfunction and weight gain. J Clin Psychiatry. 2003;64(Suppl 18):20–4. 14700451

[25] Haslam C, Brown S, Atkinson S. Patients’ experiences of medication for anxiety and depression: effects on working life. Fam Pract. 2004;21(2):204–12. 10.1093/fampra/cmh218. 15020393

[26] Loerinc AG, Meuret AE, Twohig MP, et al. Response rates for CBT for anxiety disorders: need for standardized criteria. Clin Psychol Rev. 2015;42:72–82. 10.1016/j.cpr.2015.08.004. 26319194

[27] Hofmann SG, Smits JA. Cognitive-behavioral therapy for adult anxiety disorders: a meta-analysis of randomized placebo-controlled trials. J Clin Psychiatry. 2008;69(4):621. 10.4088/jcp.v69n0415. 18363421 PMC2409267

[28] Tolin DF. Is cognitive–behavioral therapy more effective than other therapies?: A meta-analytic review. Clin Psychol Rev. 2010;30(6):710–20. 10.1016/j.cpr.2010.05.003. 20547435

[29] Basile VT, Newton‐John T, Wootton BM. Treatment histories, barriers, and preferences for individuals with symptoms of generalized anxiety disorder. J Clin Psychol. 2024;80(6):1286–305. 10.1002/jclp.23665. 38384113

[30] Heinig I, Wittchen H-U, Knappe S. Help-seeking behavior and treatment barriers in anxiety disorders: results from a representative German community survey. Community Ment Health J. 2021;57(8):1505–17. 10.1007/s10597-020-00767-5. 33471256 PMC8531057

[31] Viana MC, Kazdin AE, Harris MG, et al. Barriers to 12-month treatment of common anxiety, mood, and substance use disorders in the World Mental Health (WMH) surveys. Int J Ment Health Syst. 2025;19(1):6. 10.1186/s13033-024-00658-2. 39924481 PMC11807321

[32] Lethbridge G, Patterson B, Van Ameringen M. Cannabis and anxiety. Marijuana and Madness. 2023.

[33] Tait M-A, Costa DSJ, Campbell R, et al. Health-related quality of life in patients accessing medicinal cannabis in Australia: the QUEST initiative results of a 3-month follow-up observational study. PLoS ONE. 2023;18(9):e0290549. 10.1371/journal.pone.0290549. 37672515 PMC10482296

[34] Krediet E, Janssen DGA, Heerdink ER, et al. Experiences with medical cannabis in the treatment of veterans with PTSD: results from a focus group discussion. Eur Neuropsychopharmacol. 2020;36:244–54. 10.1016/j.euroneuro.2020.04.009. 32576481

[35] Garcia-Romeu A, Elmore J, Mayhugh RE, et al. Online survey of medicinal cannabis users: qualitative analysis of patient-level data. Front Pharmacol. 2022;13:965535. 10.3389/fphar.2022.965535. 36147312 PMC9485457

[36] Arkell TR, Downey LA, Hayley AC, et al. Assessment of medical cannabis and health-related quality of life. JAMA Netw Open. 2023;6(5):e2312522-e. 10.1001/jamanetworkopen.2023.12522. 37159196 PMC10170337

[37] Roberts L, Sorial E, Budgeon CA, et al. Medicinal cannabis in the management of anxiety disorders: a systematic review. Psychiatry Res. 2025;350:116552. 10.1016/j.psychres.2025.116552. 40413923

[38] Moreno-Sanz G, Madiedo A, Lynskey M, et al. “Flower power”: controlled inhalation of THC-predominant cannabis flos improves health-related quality of life and symptoms of chronic pain and anxiety in eligible UK patients. Biomedicines. 2022;10(10):2576. 10.3390/biomedicines10102576. 36289837 PMC9599241

[39] Wilson HB, McGrath LM. “It’s a big added stress on top of being so ill”: the challenges facing people prescribed cannabis in the UK. Int J Drug Policy. 2023;122:104220. 10.1016/j.drugpo.2023.104220. 37806073

[40] Gething K, Erku D, Scuffham P. Medicinal cannabis and consumer vulnerability in Australia: a nexus of policy and market factors. Health Expect. 2025;28(1):e70176. 10.1111/hex.70176. 39930887 PMC11811393

[41] Skene DA, McGregor IS, Todd L, et al. Use of medicinal cannabis for epilepsy in the Australian community 2023–2024: a cross-sectional survey. J Epilepsy Res. 2025;15(1):56. 10.14581/jer.25006. 40568058 PMC12185916

[42] Arkell TR, McCartney D, McGregor IS. Medical cannabis and driving. Aust J Gen Pract. 2021;50(6):357–62. 10.31128/AJGP-02-21-5840. 34059836

[43] Australia GoW. Road Traffic Act 1974 (Western Australia). Western Australia1974.

[44] Cahill SP, Lunn SE, Diaz P, et al. Evaluation of patient-reported safety and efficacy of cannabis from a survey of medical cannabis patients in Canada. Front Public Health. 2021;9:626853. 10.3389/fpubh.2021.626853. 34095048 PMC8172603

[45] Sandelowski M. What’s in a name? Qualitative description revisited. Res Nurs Health. 2010;33(1):77–84. 10.1002/nur.20362. 20014004

[46] Sandelowski M. Whatever happened to qualitative description? Res Nurs Health. 2000;23(4):334–40. 10.1002/1098-240x(200008)23:4<334::aid-nur9>3.0.co;2-g. 10940958

[47] Tong A, Sainsbury P, Craig J. Consolidated criteria for reporting qualitative research (COREQ): a 32-item checklist for interviews and focus groups. Int J Qual Health Care. 2007;19(6):349–57. 10.1093/intqhc/mzm042. 17872937

[48] Campbell S, Greenwood M, Prior S, et al. Purposive sampling: complex or simple? Research case examples. J Res Nurs. 2020;25(8):652–61. 10.1177/1744987120927206. 34394687 PMC7932468

[49] Palinkas LA, Horwitz SM, Green CA, et al. Purposeful sampling for qualitative data collection and analysis in mixed method implementation research. Adm Policy Ment Health. 2015;42(5):533–44. 10.1007/s10488-013-0528-y. 24193818 PMC4012002

[50] Chen K-F, Colantuoni E, Siddiqi F, et al. Repeated attempts using different strategies are important for timely contact with study participants. J Clin Epidemiol. 2011;64(10):1144–51. 10.1016/j.jclinepi.2010.08.007. 21109398 PMC3116960

[51] Malterud K, Siersma VD, Guassora AD. Sample size in qualitative interview studies: guided by information power. Qual Health Res. 2016;26(13):1753–60. 10.1177/1049732315617444. 26613970

[52] Braun V, Clarke V. To saturate or not to saturate? Questioning data saturation as a useful concept for thematic analysis and sample-size rationales. Qual Res Sport Exerc Health. 2021;13(2):201–16. 10.1080/2159676X.2019.1704846.

[53] Qualtrics. Qualtrics XM Platform [Computer Software]. 2024.

[54] Braun V, Clarke V. Using thematic analysis in psychology. Qual Res Psychol. 2006;3(2):77–101. 10.1191/1478088706qp063oa.

[55] QSR International Pty Ltd. NVivo qualitative data analysis software. 2012.

[56] Lincoln YS, Guba EG. Naturalistic inquiry. Newberry Park. 1985.

[57] Shenton AK. Strategies for ensuring trustworthiness in qualitative research projects. Educ Inf. 2004;22(2):63–75. 10.3233/EFI-2004-22201.

[58] Li A, Erridge S, Holvey C, et al. UK medical cannabis registry: a case series analyzing clinical outcomes of medical cannabis therapy for generalized anxiety disorder patients. Int Clin Psychopharmacol. 2024;39(6):350–60. 10.1097/YIC.0000000000000536. 38299624 PMC11424060

[59] Bapir L, Erridge S, Nicholas M, et al. Comparing the effects of medical cannabis for chronic pain patients with and without co-morbid anxiety: a cohort study. Expert Rev Neurother. 2023;23(3):281–95. 10.1080/14737175.2023.2181696. 36803620

[60] Sachedina F, Chan C, Damji RS, et al. Medical cannabis use in Canada and its impact on anxiety and depression: a retrospective study. Psychiatry Res. 2022;313:114573. 10.1016/j.psychres.2022.114573. 35598566

[61] Souza JDS, Zuardi AW, Guimarães FS, et al. Maintained anxiolytic effects of cannabidiol after treatment discontinuation in healthcare workers during the COVID-19 pandemic. Front Pharmacol. 2022;13:856846. 10.3389/fphar.2022.856846. 36263136 PMC9574068

[62] Weiss JH, Tervo-Clemmens B, Potter KW, et al. The Cannabis Effects Expectancy Questionnaire–Medical (CEEQ-M): preliminary psychometric properties and longitudinal validation within a clinical trial. Psychol Assess. 2023;35(8):659. 10.1037/pas0001244. 37289502 PMC10527809

[63] Gladis MM, Gosch EA, Dishuk NM, et al. Quality of life: expanding the scope of clinical significance. J Consult Clin Psychol. 1999;67(3):320. 10.1037/0022-006x.67.3.320. 10369052

[64] Connell J, Brazier J, O’Cathain A, et al. Quality of life of people with mental health problems: a synthesis of qualitative research. Health Qual Life Outcomes. 2012;10(1):138. 10.1186/1477-7525-10-138. 23173689 PMC3563466

[65] Reeve E, Wolff JL, Skehan M, et al. Assessment of attitudes toward deprescribing in older Medicare beneficiaries in the United States. JAMA Intern Med. 2018;178(12):1673–80. 10.1001/jamainternmed.2018.4720. 30326004 PMC6583614

[66] Law R, Gupta N, Langford AV, et al. Attitudes of Australian patients receiving inpatient mental health care towards deprescribing: a cross-sectional survey. BMC Psychiatry. 2025;25(1):269. 10.1186/s12888-025-06717-3. 40119329 PMC11929355

[67] Holmes HM, Todd A. The role of patient preferences in deprescribing. Clin Geriatr Med. 2017;33(2):165–75. 28364989 10.1016/j.cger.2017.01.004

[68] Berger M, Amminger GP, McGregor IS. Medicinal cannabis for the treatment of anxiety disorders. Aust J Gen Pract. 2022;51(8):586–92. 10.31128/AJGP-04-21-5936. 35908759

[69] Meier MH, Caspi A, Knodt RA, et al. Long-term cannabis use and cognitive reserves and hippocampal volume in midlife. Am J Psychiatry. 2022;179(5):362–74. 35255711 10.1176/appi.ajp.2021.21060664 PMC9426660

[70] Gowin JL, Ellingson JM, Karoly HC, et al. Brain function outcomes of recent and lifetime cannabis use. JAMA Netw Open. 2025;8(1):e2457069-e. 10.1001/jamanetworkopen.2024.57069. 39874032 PMC11775743

[71] Baglioni C, Nanovska S, Regen W, et al. Sleep and mental disorders: a meta-analysis of polysomnographic research. Psychol Bull. 2016;142(9):969. 10.1037/bul0000053. 27416139 PMC5110386

[72] Kahn M, Sheppes G, Sadeh A. Sleep and emotions: bidirectional links and underlying mechanisms: psychophysiology and cognitive neuroscience of sleep and sleep disorders. Int J Psychophysiol. 2013;89(2):218–28. 10.1016/j.ijpsycho.2013.05.010. 23711996

[73] Chellappa SL, Aeschbach D. Sleep and anxiety: from mechanisms to interventions. Sleep Med Rev. 2022;61:101583. 10.1016/j.smrv.2021.101583. 34979437

[74] Alvaro PK, Roberts RM, Harris JK. A systematic review assessing bidirectionality between sleep disturbances, anxiety, and depression. Sleep. 2013;36(7):1059–68. 23814343 10.5665/sleep.2810 PMC3669059

[75] de Lijster JM, Dieleman GC, Utens EM, et al. Social and academic functioning in adolescents with anxiety disorders: a systematic review. J Affect Disord. 2018;230:108–17. 10.1016/j.jad.2018.01.008. 29407534

[76] Malivoire BL, Mutschler C, Monson CM. Interpersonal dysfunction and treatment outcome in GAD: a systematic review. J Anxiety Disord. 2020;76:102310. 10.1016/j.janxdis.2020.102310. 33002755

[77] Reid M. Troubling claims of normalization: continuing stigmas within Michigan’s medical cannabis community. Deviant Behav. 2022;43(9):1068–87. 10.1080/01639625.2021.1953947.

[78] Hulaihel A, Gliksberg O, Feingold D, et al. Medical cannabis and stigma: a qualitative study with patients living with chronic pain. J Clin Nurs. 2023;32(7–8):1103–14. 10.1111/jocn.16340. 35488381

[79] Sinclair J, Abbott J, Mikocka-Walus A, et al. ‘A glimmer of hope’: perceptions, barriers, and drivers for medicinal cannabis use amongst Australian and New Zealand people with endometriosis–a qualitative study. Reprod Fertil. 2023;4:4. 10.1530/RAF-23-0049. PMC10692678 37855429

[80] Marcotte TD, Umlauf A, Grelotti DJ, et al. Driving performance and cannabis users’ perception of safety: a randomized clinical trial. JAMA Psychiat. 2022;79(3):201–9. 10.1001/jamapsychiatry.2021.4037. PMC8792796 35080588

[81] Manning B, Arkell TR, Hayley AC, et al. A semi-naturalistic open-label study examining the effect of prescribed medical cannabis use on simulated driving performance. J Psychopharmacol. 2024;38(3):247–57. 10.1177/02698811241229524. 38332655 PMC10944578

[82] Meda SA, Stevens MC, Boer ER, et al. A randomized, placebo-controlled, double-blind, pilot study of cannabis-related driving impairment assessed by driving simulator and self-report. J Psychopharmacol. 2025;39(4):364–72. 10.1177/02698811251324379. 40077985

[83] Victoria State Government. Medicinal Cannabis and Safe Driving Closed Circuit Track Trial. 2025.

[84] Sabmeethavorn Q, Bonomo YA, Hallinan CM. Pharmacists’ perceptions and experiences of medicinal cannabis dispensing: a narrative review with a systematic approach. Int J Pharm Pract. 2022. 10.1093/ijpp/riac005. 35225341

[85] Schmitz N, Richert L. Pharmacists and the future of cannabis medicine. J Am Pharm Assoc. 2020;60(1):207–11. 10.1016/j.japh.2019.11.007. 31870860

[86] Rafei P, Englund A, Lorenzetti V, et al. Transcultural aspects of cannabis use: a descriptive overview of cannabis use across cultures. Curr Addict Rep. 2023;10(3):458–71. 10.1007/s40429-023-00500-8.

