# Supplementary material for: Beyond symptom change: a qualitative descriptive study of reported quality of life experiences among adults prescribed medicinal cannabis for anxiety disorders
Source: Int J Clin Pharm. 2026 Jun 9;48(5):1979–90. doi: 10.1007/s11096-026-02176-2 (PMC13569566; doi:10.1007/s11096-026-02176-2)
Supplement: Supplementary file 1 [file 11096_2026_2176_MOESM1_ESM.docx]

Anxiety and Medicinal Cannabis: Patient’s experience in their first year of treatment Pre-Screening Questionnaire

**Summary** The aim of this study is to document and understand the journey that people go through during their first 6 to 12 months of using medicinal cannabis to manage anxiety disorders. Specifically, we want to know how using medicinal cannabis will affect a person’s quality of life from the perspective of individuals living with an anxiety disorder. We are seeking individuals aged 18 years or over, residing in Western Australia, who are beginning medicinal cannabis treatment. 1. Read the attached participant information form by clicking on the link below: Participant Information Form 2. Complete the questions on the next few pages. We will contact you afterwards about the next steps of your participation in the study if you are eligible and will then ask you to provide formal consent to our study.

Please note that the questions on the following few pages may be sensitive, however, we will treat this information with the strictest confidence. We will not link the information you provide back to you in any publications that arise from this research.

**Screening Questions**

Q1 Are you currently prescribed a medicinal cannabis product?

- Yes
- No

Q2 Will you be commencing treatment, with a prescribed medicinal cannabis product, within the next 3 months?

- Yes
- No

Q3 Are you at least 18 years old?

- Yes
- No

Q4 Do you have a diagnosis of Bipolar disorder?

- Yes
- No

Q5 Do you have a diagnosis of Schizophrenia?

- Yes
- No

**Demographic Questions**

Q6 What is your date of birth? (e.g. 23/02/2002)

________________________________________________________________

Q7 What is your postcode?

______________________________________________________________

Q8 What is your gender?

- Man (1) Female (6) Non-binary (incl. fluid, agender, etc) (3)
- Intersex (7)
- Prefer to self describe, I identify as (4) __________________________________________________
- Prefer not to say (5)

**Medical History Questions**

Q10 What anxiety disorder/s have you been diagnosed with? (Please select all that apply)

- General Anxiety Disorder
- Social Anxiety Disorder
- Panic Disorder
- Other (please list any diagnosed disorders) __________________________________________________

Q11 When were you first diagnosed with an anxiety disorder? (Please state the year)

Q12 How long have you been using prescribed medicinal cannabis?

- Have not begun treatment yet
- 3 months or less
- More than 3 months

Q13 What treatments have you undertaken prior to medicinal cannabis? (Please select all that apply)

- Anti-Anxiety Medication
- Anti-Depressant Medication
- Psychology (CBT [Cognitive Behavioural Therapy], Schema, EMDR [Eye Movement Desensitization Reprocessing])
- TMS (Transcranial Magnetic Stimulation)
- Physical Therapies (Yoga, Exercise Therapy)
- Cognitive Therapies (Mindfulness, Meditation)
- Alternative therapies (Naturopathy, Aromatherapy, Acupuncture)
- Other (Please list other treatments) __________________________________________________
- No prior treatments (10)

Q14 What are your current treatments alongside medicinal cannabis? (Please select all that apply)

- Anti-Anxiety Medication (1)
- Anti-Depressant Medication (8)
- Psychology (CBT [Cognitive Behavioural Therapy], Schema, EMDR [Eye Movement Desensitization Reprocessing]) (2)
- TMS (Transcranial Magnetic Stimulation) (3)
- Physical Therapies (Yoga, Exercise Therapy) (5)
- Cognitive Therapies (Mindfulness, Meditation) (6)
- Alternative therapies (Naturopathy, Aromatherapy, Acupuncture) (7)
- Other (Please list other treatments) (4) __________________________________________________
- No ongoing treatments (10)

Q15  So that we can contact you about the next stages of this study, please provide your email address in the space below.

________________________________________________________________
