# Supplementary material for: Beyond symptom change: a qualitative descriptive study of reported quality of life experiences among adults prescribed medicinal cannabis for anxiety disorders
Source: Int J Clin Pharm. 2026 Jun 9;48(5):1979–90. doi: 10.1007/s11096-026-02176-2 (PMC13569566; doi:10.1007/s11096-026-02176-2)
Supplement: Supplementary file 2 [file 11096_2026_2176_MOESM2_ESM.docx]

Diary Entry Guidelines

Today’s Date: xx

1. Please state which medicinal cannabis product(s) you are currently using.
   Please include the following details:
   - name of the ingredient (e.g. CBD, THC),
   - strength (e.g. 166.67mg/mL),
   - form (e.g. oil, capsule),
   - how much of it you use (e.g. 1 drop), and
   - how often (e.g. once every night).
2. Please describe how well you feel the medicinal cannabis has been working for your anxiety since your last diary entry / start of treatment. Provide as much details as you like, with examples, as we want to understand why you feel the medicinal cannabis has or has not worked.

   In your response, consider the effect of medicinal cannabis on your quality of life.

*E.g.*

- *how satisfied you are with the relationships in your life while being on medicinal cannabis,*
- *sense of belonging and community involvement while being on medicinal cannabis,*
- *level of comfort and security regarding your financial situation while being on medicinal cannabis,*
- *any changes to physical well-being like fitness or nutrition while being on medicinal cannabis*
