# Supplementary material for: Beyond symptom change: a qualitative descriptive study of reported quality of life experiences among adults prescribed medicinal cannabis for anxiety disorders
Source: Int J Clin Pharm. 2026 Jun 9;48(5):1979–90. doi: 10.1007/s11096-026-02176-2 (PMC13569566; doi:10.1007/s11096-026-02176-2)
Supplement: Supplementary file 3 [file 11096_2026_2176_MOESM3_ESM.docx]

Pre-Interview

- Interview set-up
  - Welcome
  - Summarise information, and ask if they have any questions
  - Set up voice recorder
  - Turn on recorder

Interview

1) **Context Questions:**
   a. What is your age?
   b. What is your occupation?
   c. When did you begin taking medicinal cannabis (MC)?
   d. Can you explain the process you had to go through to start MC?
2) **Cannabis products:**
   a. What is the MC product you are currently using?
      i. Follow up: CBD, THC, Strength, form, how much use, how often
3) **What was your quality of life like prior to taking MC?**
   a. Prompt: What was your life like before you used MC to treat your anxiety?
      i. FOLLOW UP (if participants focus on one area of life) What were other areas of your life (physical health, emotional health, social health e.g.) like?
4) **How has it been since you started taking MC?**
   a. Prompt: Have you noticed any changes in your life since beginning MC treatment
      i. FOLLOW UP: What has your sleep been like since using MC?
      ii. FOLLOW UP: (Bring in life areas from Q3) What has that been like since using MC?
5) **What has your anxiety been like since you have been on MC treatment?**
   a. Prompt: Can you describe to me what your anxiety is like since you started taking MC?
      i. FOLLOW UP: (if share positive improvement) Have you noticed any other improvements since taking MC / Can you expand on what was the improvement?
      ii. FOLLOW UP (if share negative) Can you expand on what has been negative?
      iii. FOLLOW UP: Can you describe if any, changes in your relationships since using MC
6) **What has the experience of MC been like compared to other treatments you have used in the past?**
   a. Prompt: What has it been like using MC compared to other treatments
7) **Has using MC had any impact on daily activities?**
   a. FOLLOW UP: If yes, then in what ways
   b. FOLLOW UP: (Bring in life areas from Q3) What has that been like since using MC?
8) **What are your plans to manage your anxiety moving forward?**
   a. Prompt: Do you think you will continue to use MC for your treatment?
      i. FOLLOW UP: Is there anything you wish you had known before starting MC
      ii. FOLLOW UP: (If speak about continuing treatment): why do you want to continue?
      iii. FOLLOW UP (if speak about stopping treatment): why do you want to stop?
9) **What else would you like to share about your experience and your quality of life since being on MC?**

Post Interview

1) Conclusion
   a. Highlight information on accessing findings in Participant Information Sheet
   b. Inform interviewee they have the option to review their transcript
   c. Turn off recorder
   d. Send through gift receipt
